# In Vivo PET Imaging of ^89^Zr-Labeled Natural Killer Cells and the Modulating Effects of a Therapeutic Antibody

**DOI:** 10.2967/jnumed.124.267876

**Published:** 2024-07

**Authors:** Truc T. Pham, Alicia Chenoweth, Natasha Patel, Arshiya Banu, Gabriel Osborn, Philip J. Blower, Sophia N. Karagiannis, Michelle T. Ma

**Affiliations:** 1 Department of Imaging Chemistry and Biology, School of Bioengineering and Imaging Sciences, King’s College London, London, United Kingdom;; 2 St. John’s Institute of Dermatology, School of Basic and Medical Biosciences, King’s College London, London, United Kingdom; and; 3 Breast Cancer Now Research Unit, School of Cancer and Pharmaceutical Sciences, King’s College London, Guy’s Hospital, London, United Kingdom

**Keywords:** natural killer cell, ADCC, trastuzumab, HER2 receptor, cell tracking

## Abstract

Natural killer (NK) cells can kill cancer cells via antibody-dependent cell-mediated cytotoxicity (ADCC): a tumor-associated IgG antibody binds to the Fcγ receptor CD16 on NK cells via the antibody Fc region and activates the cytotoxic functions of the NK cell. Here, we used PET imaging to assess NK cell migration to human epidermal growth factor receptor 2 (HER2)–positive HCC1954 breast tumors, examining the influence of HER2-targeted trastuzumab antibody treatment on NK cell tumor accumulation. **Methods:** Human NK cells from healthy donors were expanded ex vivo and labeled with [^89^Zr]Zr-oxine. In vitro experiments compared the phenotypic markers, viability, proliferation, migration, degranulation, and ADCC behaviors of both labeled (^89^Zr-NK) and unlabeled NK cells. Female mice bearing orthotopic human breast HCC1954 tumors were administered ^89^Zr-NK cells alongside trastuzumab treatment or a sham treatment and then scanned using PET/CT imaging over 7 d. Flow cytometry and γ-counting were used to analyze the presence of ^89^Zr-NK cells in liver and spleen tissues. **Results:** ^89^Zr cell radiolabeling yields measured 42.2% ± 8.0%. At an average specific activity of 16.7 ± 4.7 kBq/10^6^ cells, ^89^Zr-NK cells retained phenotypic and functional characteristics including CD56 and CD16 expression, viability, migration, degranulation, and ADCC capabilities. In vivo PET/CT studies indicated predominant accumulation of ^89^Zr-NK cells in the liver and spleen. Ex vivo analyses of liver and spleen tissues indicated that the administered human ^89^Zr-NK cells retained their radioactivity in vivo and that ^89^Zr did not transfer to cells of murine soft tissues, thus validating this ^89^Zr PET method for NK cell tracking. Notably, ^89^Zr-NK cells migrated to HER2-positive tumors, both with and without trastuzumab treatment. Trastuzumab treatment was associated with an increased ^89^Zr-NK cell signal at days 1 and 3 after injection. **Conclusion:** In vitro, ^89^Zr-NK cells maintained key cellular and cytotoxic functions. In vivo, ^89^Zr-NK cells trafficked to HER2-postive tumors, with trastuzumab treatment correlating with enhanced ^89^Zr-NK infiltration. This study demonstrates the feasibility of using PET to image ^89^Zr-NK cell infiltration into solid tumors.

## Visual Abstract

**Figure:**
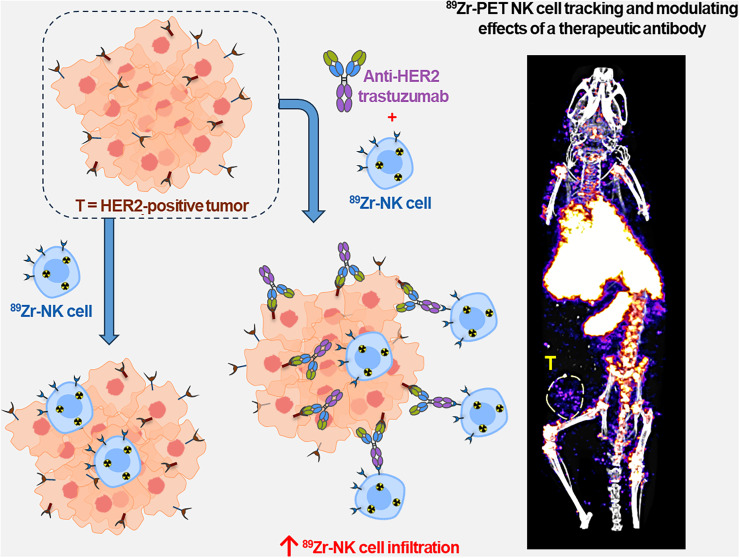

Monoclonal IgG antibodies used in clinical oncology exert therapeutic effects by inhibiting cancer cell receptors that drive tumor proliferation. In breast cancer treatment, trastuzumab targets human epidermal growth factor receptor 2 (HER2). By binding of the antibody Fab region to HER2, trastuzumab prevents HER2 dimerization, thus inhibiting the downstream proliferative signals that promote tumor growth. In addition, in combination with immune effector cells (most notably natural killer [NK] cells), trastuzumab can trigger antibody-dependent cell-mediated cytotoxicity (ADCC), resulting in immune-cell activation and cancer-cell lysis.

NK cells express the low-affinity yet potent FcγRIIIA (or CD16) activating receptor. In vivo, a specific IgG monoclonal antibody can engage via its Fab region with its target antigen on a cancer cell; simultaneously, the Fc region of the monoclonal antibody is recognized by CD16, facilitating the activation of cytotoxic NK cell functions. NK cells can also independently induce cytotoxic responses against cancer cells through lytic synapse formation or apoptotic pathways. NK cells also modulate other immune responses involving T cells, macrophages, and dendritic cells through cytokine or chemokine pathways.

ADCC can contribute to the efficacy of HER2-targeted immunotherapies. In patients administered HER2-targeted immunotherapies, improved responses are associated with higher tumor infiltration of NK cells (1–3) or lymphocytes (4,5) in HER2-positive breast cancer biopsies. Furthermore, analyses of surgical specimens from HER2-positive breast cancers have previously revealed an increase in NK cells in tumor tissue after trastuzumab treatment, relative to specimens collected either before treatment (1) or from case-matched controls who did not receive trastuzumab treatment (2). Similarly, in a murine model of HER2-positive breast cancer, an increase in NK cell numbers was observed in tumors after treatment with a trastuzumab-derived antibody–drug conjugate (6). Highlighting the clinical significance of ADCC effects mediated by NK cells, a phase 1 clinical trial in patients with HER2-positive tumors recently reported that a therapeutic regime of expanded autologous NK cells in combination with trastuzumab is safe, exhibits tumor engagement, and shows preliminary evidence of therapeutic efficacy (7). Compared with paired tumor biopsies obtained before treatment, increases in NK cells, lymphocytes, and apoptosis activity were observed in biopsies after treatment.

The distribution and tumor infiltration of NK cells, and how this may be influenced by therapeutic antibody treatment, is therefore important in understanding the immunologic landscape of cancer at the cellular, tissue, and whole-body levels. Whole-body imaging can provide spatial and longitudinal insights into the distribution of NK cells in vivo. In direct cell-tracking methods, NK cells are labeled ex vivo with a contrast agent and then administered for in vivo tracking using whole-body imaging. This approach has been previously applied using optical imaging (8,9), MRI (10), SPECT imaging or γ-scintigraphy with [^111^In]In-oxine (11–13), and PET imaging using [^89^Zr]Zr-oxine (14). Optical imaging and MRI can provide high-resolution images but lack quantitative attributes. In contrast, PET and γ-scintigraphy/SPECT imaging can provide real-time and quantitative information, and both are highly sensitive.

A recently developed method enables the radiolabeling of cells using [^89^Zr]Zr-oxine (^89^Zr half-life, 78.41 h), facilitating longitudinal cell tracking over 1–2 wk with PET (15). The method has been applied to track NK cells (14), T cells (16,17), and bone marrow cells (18) in vivo, among others (19). Similar methods using [^111^In]In-oxine are well established for ^111^In cell tracking with γ-scintigraphy/SPECT imaging. In both cases, [^89^Zr]Zr-oxine and [^111^In]In-oxine diffuse into cells and release the radionuclide intracellularly, resulting in cell labeling. Previous in vivo studies have investigated the biodistribution of ^89^Zr- and ^111^In-labeled NK cells in healthy and cancer subjects without augmentation of therapeutic adjuvants (11–14). Here, we use PET/CT tracking to study the biodistribution of ^89^Zr-labeled human NK (^89^Zr-NK) cells in mice bearing HER2-positive solid orthotopic HCC1954 human breast tumors and to assess whether administration of HER2-targeted trastuzumab enhances NK cell infiltration into tumors.

## MATERIALS AND METHODS

### Human NK Cells, ^89^Zr Radiolabeling, and Cell Assays

Experiments using human blood received approval from King’s College London–Research Ethics Committee (study reference HR/DP-20/21-24483). All donors provided written informed consent. NK cells were isolated from the mononuclear cell layer of human peripheral blood, cultured, and expanded ex vivo (20). [^89^Zr]Zr-oxine (∼45 kBq/10^6^ cells) was added to ex vivo–expanded NK cells suspended in phosphate-buffered saline (PBS) at 15 × 10^6^ to 20 × 10^6^ cells/mL, followed by incubation for 15 min at ambient temperature (15), as shown in the supplemental materials (supplemental materials are available at http://jnm.snmjournals.org).

Cell retention, viability and growth assays, chemotaxis assays, CD107a degranulation assays, and ADCC assays were performed on ^89^Zr-NK and unlabeled NK cells (supplemental materials).

### In Vivo Murine and PET/CT Biodistribution Studies

Animal experiments were ethically reviewed by the Animal Welfare and Ethical Review Board at King’s College London and were performed in accordance with the Animals (Scientific Procedures) Act 1986 U.K. Home Office regulations governing animal experimentation. NSG mice (8-to-10-wk-old female NOD-scid-γ [NOD.Cg-Prkdc^scid^ Il2rg^tm1WjI^/SzJ]; Charles River) were inoculated with 1.5 × 10^6^ HCC1954 cells in the left mammary fat pad between the fourth and fifth pairs of nipples. Experiments commenced when tumors reached 100–150 mm^3^.

Tumor-bearing mice were randomized into 3 groups and intravenously administered 1 × 10^7^ freshly radiolabeled NK cells (150–200 kBq), rhIL-15 (2,500 IU), and either PBS, anti–normal immunosuppressive protein isotype control (5 mg/kg), or trastuzumab (5 mg/kg) (∼200 μL of PBS). NK cells from 3 healthy human volunteers were used. Additional doses of rhIL-15 (2,500 IU/dose) were given on days 3 and 6 via intraperitoneal injection to support the in vivo survival and expansion of NK cells (13).

PET/CT imaging was conducted using a nanoScan PET/CT scanner (Mediso) on days 1, 3, and 7 after cell injection. The images were coregistered and analyzed using VivoQuant version 3.0 (Invicro). SPECT/CT imaging using [^111^In]In-CHX-A″-DTPA-trastuzumab was performed to determine the antibody biodistribution (supplemental materials).

### Ex Vivo Flow Cytometry Study

Single-cell suspensions, prepared from mouse liver and spleen collected 3 d after ^89^Zr-NK cell administration, were stained with antihuman antibodies CD56-FITC, CD16-APC, and CD45-PE-Cy7. CD45-positive and CD45-negative populations were sorted on a BD FACSMelody cell sorter (BD Biosciences) and collected for γ-counting (supplemental materials).

### Statistical Analysis

Independent experiments were conducted on separate days using NK cells from different donors. Statistical analysis was conducted using Prism 9.5.0 (GraphPad Software). Data are presented as mean ± SD. Statistical significance was determined using either an unpaired or paired 2-tailed Student *t* test. For tumor uptake analysis across the treatment groups, 1-way ANOVA followed by *t* tests with multiple comparison correction (Tukey method) was performed. A *P* value below 0.05 was considered statistically significant.

## RESULTS

### In Vitro–Labeled ^89^Zr-NK Cells Show Comparable Phenotype and Functional Characteristics to Unlabeled NK Cells

Using a prefabricated oxine kit (15), [^89^Zr]Zr-oxine was reproducibly synthesized, with a radiochemical yield of 90.0% ± 5.8%. Ex vivo–expanded human primary NK cells from peripheral blood were incubated with [^89^Zr]Zr-oxine at room temperature for 15 min, with cell radiolabeling efficiencies of 42.2% ± 8.0%. The final specific activity measured 16.7 ± 4.7 kBq/10^6^ cells.

**FIGURE 1.**
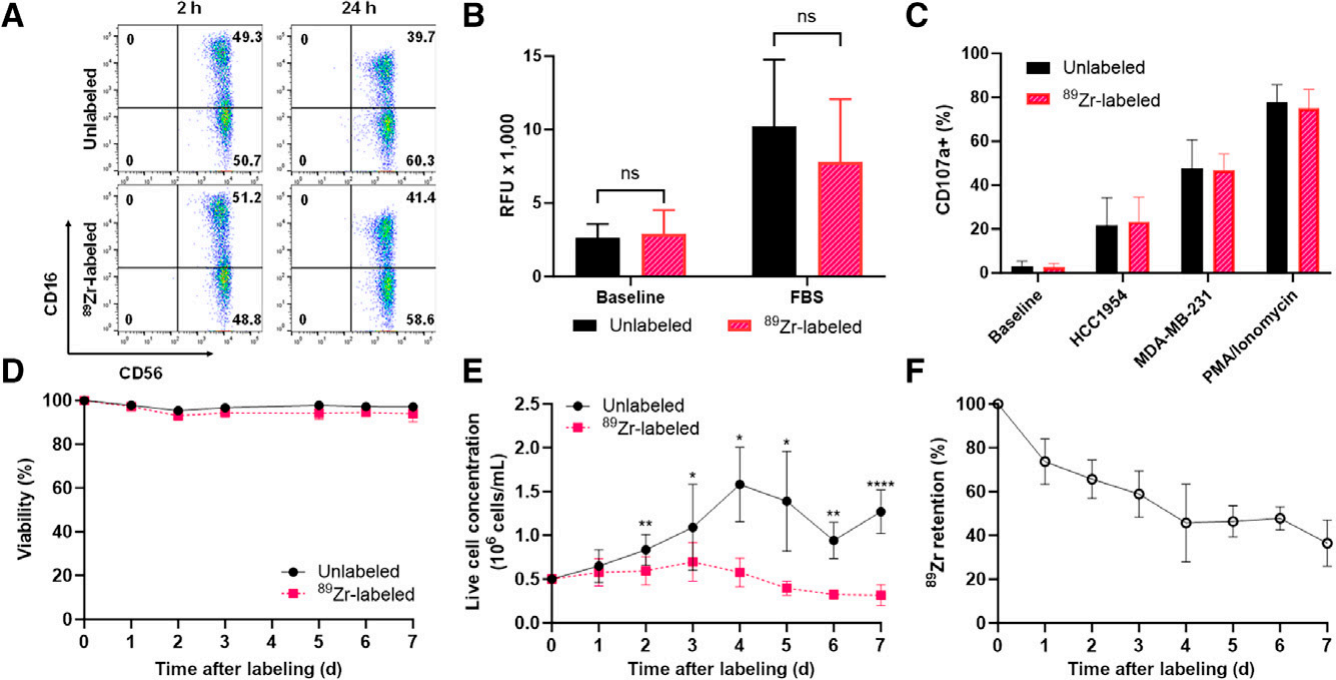
In vitro functional and phenotypic characteristics of ^89^Zr-NK cells and ^89^Zr-retention. (A) Flow cytometry shows comparable CD56 and CD16 expression in unlabeled and ^89^Zr-NK cells. (B) Chemotaxis assays show that chemotactic responses to fetal bovine serum, in unlabeled and ^89^Zr-NK cells, are similar (mean ± SD, *n* = 5). (C) Degranulation assays reveal similar degranulation levels in ^89^Zr-NK and unlabeled NK cells under various conditions (mean ± SD, *n* = 4). (D and E) Viability (D) and proliferation profiles (E) indicate that both ^89^Zr-NK and unlabeled NK cells remained viable for up to 7 d in culture without interleukins. Although unlabeled NK cells continued to proliferate, ^89^Zr-NK cells did not (mean ± SD, *n* = 7). (F) ^89^Zr retention in ^89^Zr-NK cells gradually decreased over 7 d in culture, with 36.4% ± 10.5% of initial activity remaining on day 7 (mean ± SD, *n* = 7). *P < 0.05. **P < 0.01. ****P < 0.0001. FBS = fetal bovine serum; ns = nonsignificant; PMA = phorbol 12-myristate-13-acetate; RFU = relative fluorescence units.

To assess the effect of ^89^Zr labeling on human NK cells, several NK cell markers and functions were measured in ^89^Zr-NK cells, including phenotypic CD56 and CD16 expression and functional characteristics, namely, migratory ability, viability and proliferation, and cytotoxic degranulation and ADCC responses. Flow cytometry of both ^89^Zr-NK (measured 2 and 24 h after radiolabeling) and unlabeled NK cells indicated that CD56 and CD16 expression was unaffected by ^89^Zr labeling (Fig. 1A). The migration of ^89^Zr-NK (24 h after radiolabeling) and unlabeled NK cells toward fetal bovine serum stimulus was similar, with migration toward fetal bovine serum for both cells shown to be 3- to 4-fold higher than background migration (Fig. 1B). The cytotoxic response of NK cells was measured in a degranulation assay by quantifying the levels of lysosome-associated membrane protein-1 (CD107a) (Fig. 1C). The percentages of CD107a-positive cells in both ^89^Zr-NK cells and unlabeled NK cells were comparable across all conditions: high percentages in the phorbol 12-myristate-13-acetate/ionomycin–positive controls (74.9% ± 8.7% and 77.8% ± 8.0%), moderate percentages in the presence of either HCC1954 (23.4% ± 11.0% and 21.5% ± 12.7%) or MDA-MB-231 (46.7.4% ± 7.4% and 47.6% ± 13.0%) cocultures, and minimal baseline degranulation (2.4% ± 1.9% and 2.9% ± 2.4%).

The viability of ^89^Zr-NK cells in culture, determined by trypan blue assay, remained unaffected (>95%) over 7 d, as compared with unlabeled NK cells (Fig. 1D). However, in the absence of interleukins, ^89^Zr-NK cells did not proliferate after radiolabeling, even at low levels of associated ^89^Zr (∼16 kBq/10^6^ cells) (Fig. 1E), whereas unlabeled NK cells continued to proliferate; the difference became significant from day 2 in culture (*P* < 0.05). Lastly, ^89^Zr-radioactivity slowly dissociated from NK cells, with the initial activity in the cells remaining at 73.7% ± 10.4% at day 1, 58.9% ± 10.5% at day 3, and 36.4% ± 10.5% at day 7 (Fig. 1F).

**FIGURE 2.**
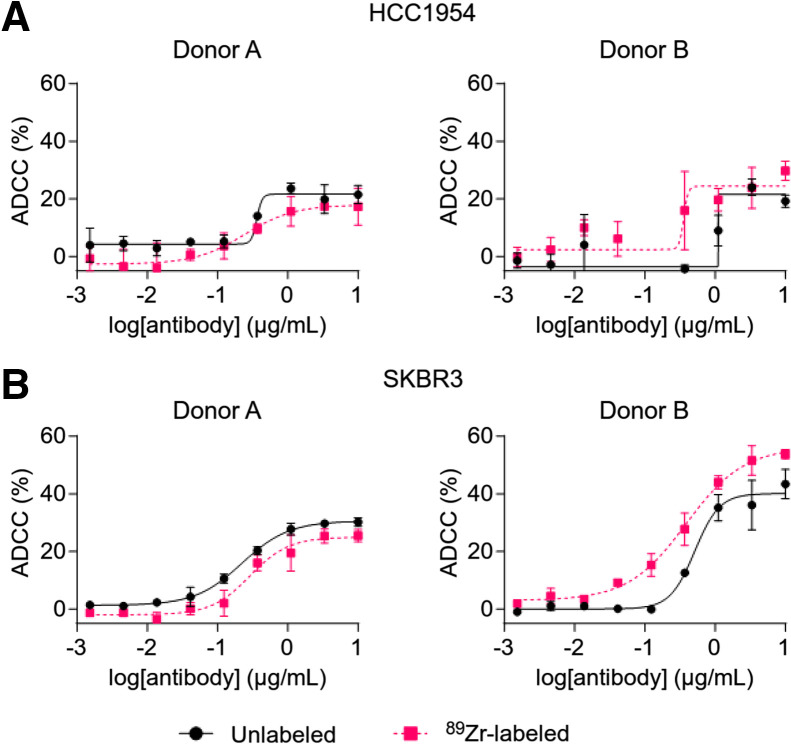
ADCC assays using ^89^Zr-labeled and unlabeled NK cells against HER2-expressing breast cancer cell lines HCC1954 (A) and SKBR3 (B) (10:1 effector/target ratio) and various trastuzumab concentrations. ^89^Zr-NK cells demonstrated similar ADCC response to unlabeled NK cells. ADCC response varied among human NK cells from different healthy volunteers (Supplemental Fig. 1). Data were fitted to 4-parameter logistic curve.

The cytolytic activity of ^89^Zr-NK cells (24 h after radiolabeling) and unlabeled NK cells, from 4 healthy human donors, was assessed in ADCC assays using trastuzumab and HER2-positive breast cancer cell lines SKBR3 and HCC1954 (Fig. 2). For each donor, ^89^Zr-NK cells displayed ADCC effects highly similar to those with unlabeled NK cells. Consistent with published literature, ADCC effects were donor-specific: trastuzumab boosted the cytolytic activity of NK cells from donors A and B against both cell lines in a concentration-dependent manner, whereas negligible ADCC effects were observed for donors C and D under our experimental conditions (Supplemental Fig. 1).

### ^89^Zr-NK Cells in HCC1954 Tumor–Bearing Mice Demonstrate Enhanced Tumor Localization with Trastuzumab Treatment

Using PET/CT imaging, the migration and accumulation of human ^89^Zr-NK cells were studied in female NSG immunodeficient mice (which lack T, B, and NK cells) bearing orthotopic human HCC1954 breast tumors. Despite expressing high levels of HER2, HCC1954 cells are resistant to the Fab-mediated HER2-binding downstream inhibitory (and associated therapeutic) effects of trastuzumab (21). Therefore, the HCC1954 model is useful in assessing trastuzumab treatment on NK cell tumor accumulation in a setting where the antibody can only exert Fc-mediated effector functions against these tumors.

Here, 1 × 10^7 89^Zr-NK cells were coadministered to mice intravenously (tail vein) in combination with either a PBS sham (*n* = 6), HER2-targeted trastuzumab (*n* = 6), or a hapten-specific anti–normal immunosuppressive protein IgG1 isotype control antibody (*n* = 4) (which does not recognize mammalian antigens including HER2 but bears a human Fc region capable of binding to CD16 receptors of NK cells). NK cells from 3 different healthy volunteers were used, with 2 animals in each group receiving ^89^Zr-NK cells from each volunteer. Additionally, mice received intraperitoneal doses of rhIL-15 to support the survival of NK cells in vivo (13). PET/CT scanning was performed 1, 3, and 7 d after injection of ^89^Zr-NK cells.

**FIGURE 3.**
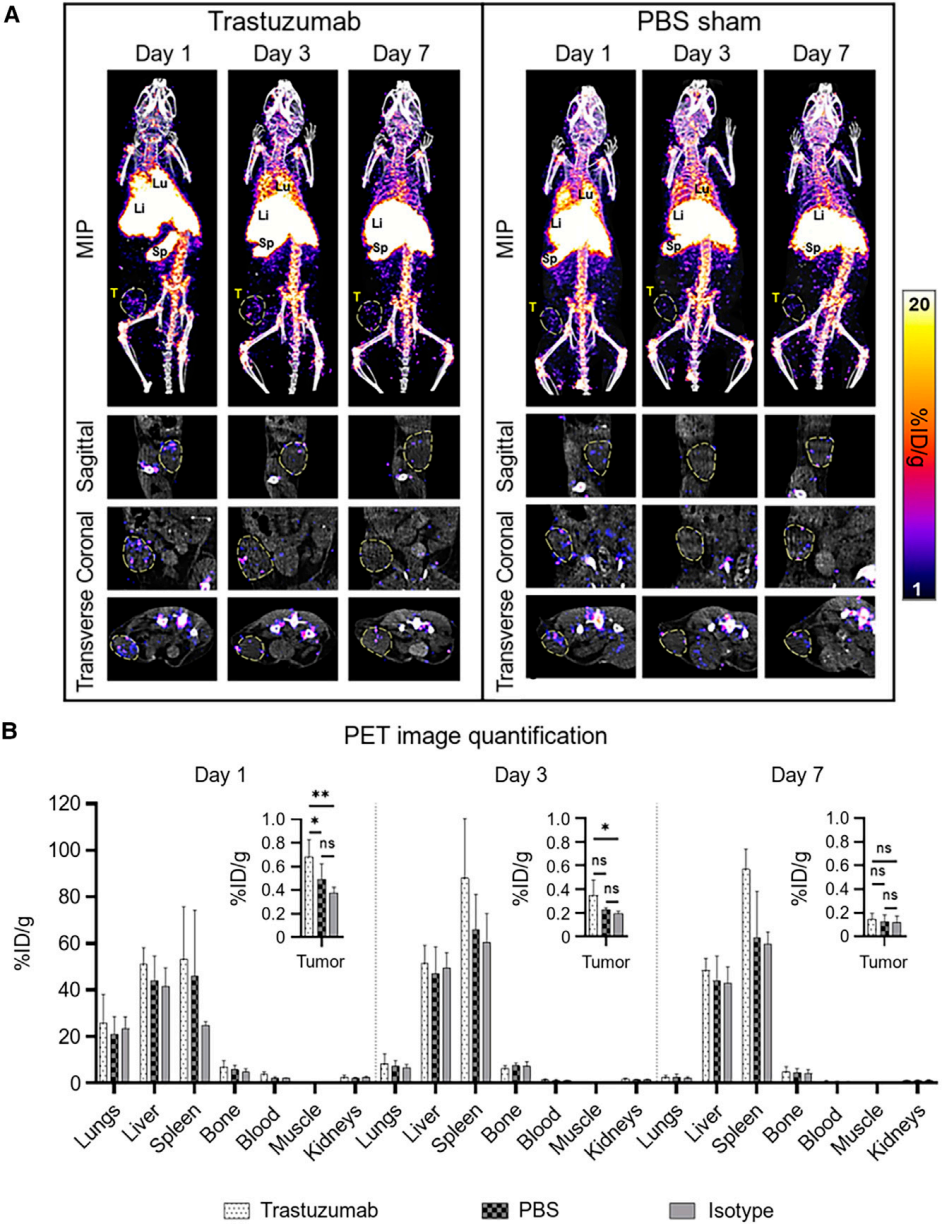
Biodistribution of ^89^Zr-NK cells in female NSG mice bearing orthotopic HER2-expressing HCC1954 tumors. (A) Representative maximum-intensity projection (MIP) and tumor slice PET/CT images of mice administered ^89^Zr-NK cells (10^7^ cells, ∼150–200 kBq) in combination with trastuzumab (5 mg/kg) or PBS only. Tumors are outlined for clarity. (B) PET image quantification of selected organs and tumors (mean ± SD, *n* = 4–6/group). *P < 0.05. **P < 0.01. Li = liver; Lu = lungs; MIP = maximum-intensity projection; ns = nonsignificant; Sp = spleen; T = tumor.

In all mice, ^89^Zr-NK cells migrated to the lungs, liver, and spleen within the first 24 h, with redistribution from the lungs to the liver and spleen over 7 d (Fig. 3A).

PET/CT imaging indicated that ^89^Zr-NK cells accumulated in tumors but decreased from day 1 to day 7 after injection. ^89^Zr-NK cell tumor distribution was highly heterogeneous in all groups of mice. A significant proportion of ^89^Zr-NK cells localized at the periphery of tumors (Supplemental Video 1). Importantly, from PET quantification (Fig. 3B), mice in the trastuzumab-treated group demonstrated significantly higher ^89^Zr-NK cell infiltration in tumors at 1 d after injection (0.66 ± 0.13 percentage of injected dose [%ID]·g^−1^) compared with both the sham group (0.38 ± 0.16 %ID·g^−1^, *P* = 0.0063) and the isotype group at 1 d after injection (0.37 ± 0.11 %ID·g^−1^, *P* = 0.0499). Similarly, at 3 d after injection, the trastuzumab-treated group demonstrated higher ^89^Zr-NK cell tumor infiltration (0.34 ± 0.12 %ID·g^−1^) compared with both the sham group (0.21 ± 0.04 %ID·g^−1^, *P* = 0.0593) and the isotype group (0.18 ± 0.03 %ID·g^−1^, *P* = 0.0268). At the same time points, there was no significant difference in tumor activity between the isotype-treated group and the PBS sham–treated group. In concordance with the gradual loss of the ^89^Zr label observed after culturing ^89^Zr-NK cells for 7 d in vitro, in this in vivo study, only low amounts of ^89^Zr were detected in tumors 7 d after injection (0.15 ± 0.04 %ID·g^−1^ in the trastuzumab group) and no differences between groups were found at this time point (Fig. 3B).

Ex vivo biodistribution and tissue γ-counting experiments at days 3 and 7 provided results similar to those of PET image quantification, demonstrating comparable radioactivity concentrations in major organs and tissues (Supplemental Fig. 2). However, no significant differences in tumor radioactivity between trastuzumab-treated, PBS sham, and isotype groups were found at day 3, likely because of the loss of NK cells during washing steps after dissection, given their predominant localization in the tumor periphery.

### ^89^Zr-NK Cells Accumulate and Persist in Spleen and Liver Tissues

**FIGURE 4.**
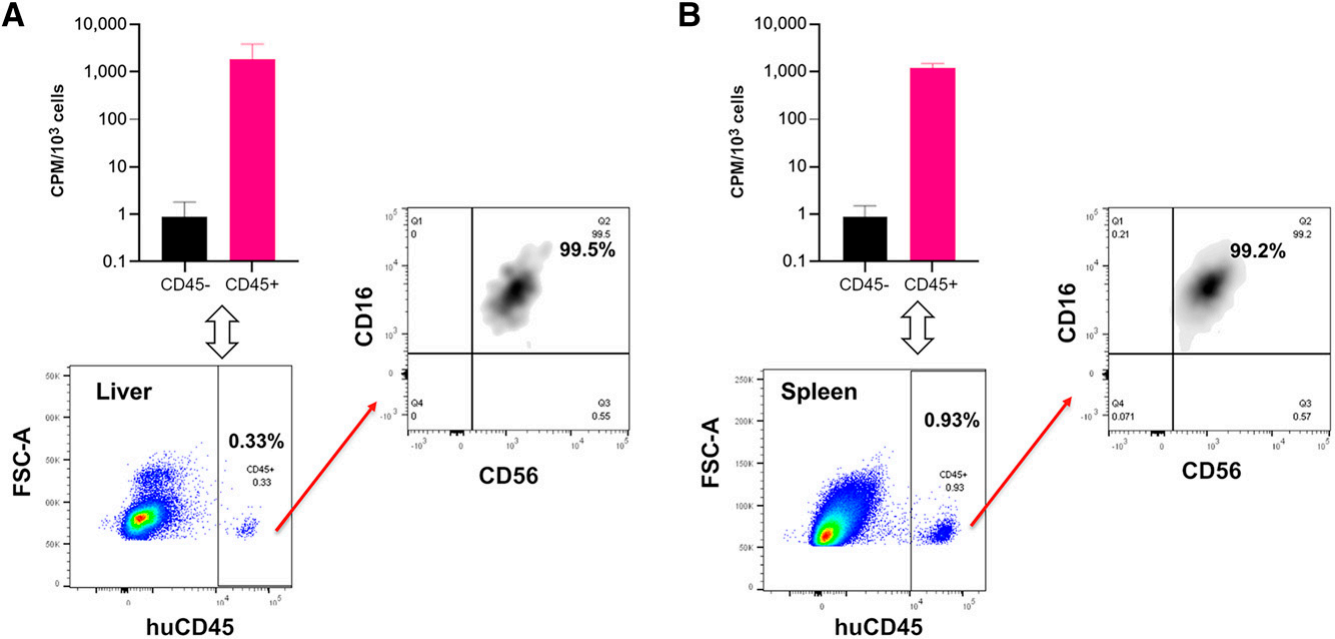
Flow cytometry and γ-counting of CD45-positive and CD45-negative cell populations from murine liver (A) and spleen (B) revealed that ^89^Zr radioactivity was associated with human CD45-positive (huCD45) cells (*n* = 4, mean ± SD), which were confirmed as C56-positive/CD16-positive NK cells. CPM = counts per minute; FSC-A = forward scatter area.

To validate this ^89^Zr-NK PET imaging method, ex vivo flow cytometric phenotyping was undertaken. Liver and spleen tissues from mice administered ^89^Zr-NK cells (3 d after injection) were processed to form single-cell suspensions, followed by staining. Flow cytometry (Fig. 4) revealed the presence of human CD45-positive cells, which were further identified as CD56-positive/CD16-positive NK cells. CD45-positive and CD45-negative cell populations were separated and counted for radioactivity: human CD45-positive cells from the liver measured an average of 1,899 counts per minute/10^3^ cells, whereas CD45-negative cells measured 0.9 counts per minute/10^3^ cells; CD45-positive cells from the spleen measured 1,230 counts per minute/10^3^ cells, whereas CD45-negative cells measured 0.8 counts per minute/10^3^ cells. This indicated that the ^89^Zr signal was largely associated with human NK cells in soft tissue.

**FIGURE 5.**
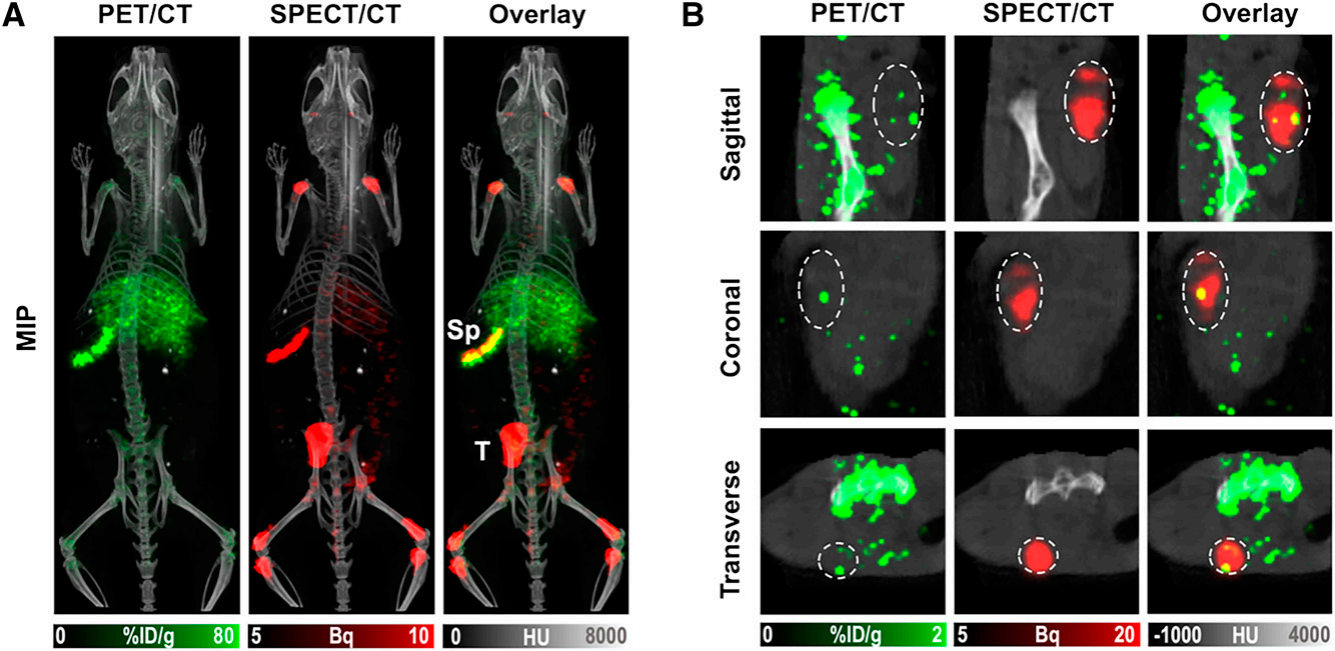
Maximum-intensity projection (MIP) (A) and tumor slice PET/CT and SPECT/CT (B) images of mice 3 d after injection of ^89^Zr-NK cells in combination with [^111^In]In-CHX-A″-DTPA-trastuzumab. HU = Hounsfield unit; Sp = spleen; T = tumor.

The accumulation of ^89^Zr-NK cells was particularly high in the spleen across all groups, with ^89^Zr radioactivity concentration the highest in animals coadministered trastuzumab at 3 and 7 d after injection (Fig. 3B). To investigate this further, ^89^Zr-NK cells were coadministered with the [^111^In]In-CHX-A″-DTPA-trastuzumab immunoconjugate. At 3 d after injection, PET/CT and SPECT/CT showed colocalization of the ^89^Zr signal and ^111^In signal (0.45 ± 0.001 %ID) in splenic tissue (Fig. 5A). SPECT/CT imaging also indicated that significant amounts of [^111^In]In-CHX-A″-DTPA-trastuzumab accumulated in HER2-positive HCC1954 tumors (2.41 ± 0.006 %ID). Importantly, the heterogeneous PET signal, attributed to infiltration of ^89^Zr-NK cells, was coincident with the SPECT signal of [^111^In]In-CHX-A″-DTPA-trastuzumab in tumor tissue (Fig. 5B).

**FIGURE 6.**
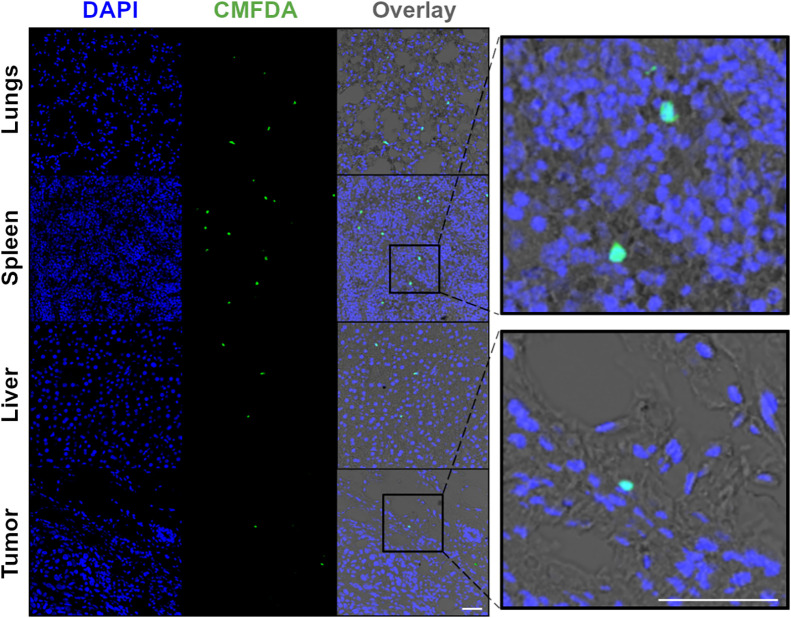
Confocal microscopy images (×40 magnification) of lung, spleen, liver, and tumor sections 3 d after injection of NK cells and trastuzumab. Sections stained with DAPI revealed presence of CMFDA–labeled NK cells. Scale bar is 50 μm. CMFDA = 5-chloromethylfluorescein diacetate; DAPI = 4′,6-diamidino-2-phenylindole.

Lastly, to confirm the presence of human NK cells in spleen, liver, and tumors in this specific NSG orthotopic HCC1954 breast cancer murine model, NK cells were labeled ex vivo with fluorescent CMFDA (5-chloromethylfluorescein diacetate), before intravenous in vivo administration, both with and without trastuzumab. Confocal microscopy of tumor, lung, spleen, and liver sections obtained 3 d after injection and costained with DAPI (4′,6-diamidino-2-phenylindole) revealed the presence of CMFDA-labeled NK cells in these tissues (Fig. 6; Supplemental Fig. 3).

## DISCUSSION

^89^Zr-oxine (19) has been increasingly used for PET tracking of immune cells, including NK, chimeric antigen receptor T, γδ-T, and bone marrow cells (16,17). Here, the ^89^Zr radiolabeling yields and in vitro cellular retention of NK cells were comparable to those of previous studies of NK cells (14) and other immune cells (17). Importantly, the average specific activity of ^89^Zr-NK cells used here did not significantly alter crucial functional NK cell characteristics, including NK cellular viability, motility, activation, and cytolytic potency, and key phenotypic markers, consistent with prior reports (14). Although in vitro retention of ^89^Zr radioactivity decreased over 7 d, retention at days 1 and 3 was sufficiently high to enable reliable in vivo ^89^Zr-NK cell PET tracking.

PET/CT images revealed initial margination of ^89^Zr-NK cells in lung—the first capillary bed encountered by intravenously injected cells—followed by redistribution to the liver and spleen. This is consistent with many prior cell-tracking reports (Supplemental Table 1) (11–14).

Additionally, ^89^Zr activity accumulated in the mouse bones, most prominently in the joints, consistent with reports that dissociated oxyphilic Zr^4+^ is associated with regions of high bone mineralization (22). It is highly probable that a portion of this signal is a result of NK cell migration to the bone marrow (11). In addition, our ex vivo flow cytometric evaluations demonstrated that NK cells isolated from liver and spleen tissues showed 1,500-fold and 2,100-fold higher levels of ^89^Zr radioactivity than did murine spleen and liver cells, respectively, indicating that in soft tissue, ^89^Zr activity is largely associated with only the administered human NK cells.

Prior imaging studies in murine cancer models and cancer patients, showing the migration of prelabeled NK cells to tumors, are consistent with our data. Near-infrared optical imaging has indicated the in vivo migration of near-infrared-dye–labeled human NK cells to human MDA-MB-231 breast tumors in NSG mice (8). Whole-body SPECT imaging studies in patients with renal cell carcinoma have evidenced that ^111^In-labeled allogenic NK cells migrate to metastases (11).

We show that ^89^Zr-NK cells migrate to HCC1954 breast cancer xenografts, with or without trastuzumab treatment. At 1 and 3 d after injection, enhanced NK cell infiltration in tumor tissue is associated with coadministration of trastuzumab, aligning with clinical evidence (1,2,7). However, although statistically significant, this enhancement remains relatively modest. Several mechanisms could restrict NK cell tumor infiltration. In solid tumors, such as ovarian carcinoma and lung cancer, NK cell activation by antitumor antibody therapy is limited (23–25). NK cells may be exhausted in the tumor microenvironment because of the downregulation of activation markers (26), leading to reduced infiltration and retention. Alternatively, on activation, CD16 can be shed or sequestered, thus modulating ADCC effects (27).

Animals coadministered trastuzumab demonstrated higher splenic uptake of ^89^Zr-NK cells than did animals coadministered a PBS sham at 3 and 7 d after injection. SPECT/CT indicated that significant amounts of [^111^In]In-CHX-A″-DTPA-trastuzumab also localized to the spleen. Splenic vasculature is highly perfused and permeable to many blood-borne components: immune cells (11–14) and IgG antibodies (28) are well documented to accumulate in splenic tissue. In immune-deficient NSG mice that lack B cells and therefore normal endogenous levels of circulating immunoglobulins, exogenous human IgG antibodies exhibit particularly high uptake in the spleen. This has been attributed to antibody Fc binding to unoccupied murine Fc receptors expressed on spleen-residing monocytes, neutrophils, macrophages, and dendritic cells (29). In this study, it is possible that high residency of trastuzumab antibody in the spleen increases the accumulation of human ^89^Zr-NK cells, which can compete with endogenous immune cells for binding to the humanized trastuzumab Fc region. However, we note that animals coadministered an IgG isotype control alongside ^89^Zr-NK cells did not show the same levels of splenic ^89^Zr activity as did animals coadministered trastuzumab.

Multiple doses of NK cells combined with antibody treatment can augment ADCC effects better than a single dose of NK cells or multiple doses of antibody alone (27). Future studies of different dosing regimens of ^89^Zr-NK cells and antibody will allow more in-depth studies of cellular dynamics in the context of therapy.

## CONCLUSION

We have shown the utility of [^89^Zr]Zr-oxine for radiolabeling and tracking of human NK cells in a murine orthotopic human breast cancer model. This sensitive method enables quantitative assessment of changes in NK cell biodistribution in response to antibody therapies. Importantly, our findings reveal that NK cells migrate to orthotopic HER2-expressing HCC1954 tumors, with enhanced infiltration facilitated by HER2-targeted trastuzumab at early time points, aligning with clinical evidence. The use of ^89^Zr and PET/CT can therefore aid the development and understanding of antibody therapies, in the context of the immune environment and Fc-mediated therapeutic effects.

## DISCLOSURE

This research was supported by Cancer Research U.K. (C30122/A11527; C30122/A15774; C4278/A27066) including a Career Establishment Award (C63178/A24959), the EPSRC (EP/S032789/1), Wellcome Trust (WT212885/Z/18/Z; WT201959/Z/16/Z; WT088641/Z/09/Z), Breast Cancer Now (147; KCL-BCN-Q3), and the MRC (MR/L023091/1, MR/N013700/1). Philip Blower has submitted a patent application related to [^89^Zr]Zr-oxine technology. Sophia Karagiannis is a founder and shareholder of Epsilogen Ltd. and declares patents on antibody technologies. No other potential conflict of interest relevant to this article was reported.

## Notes

KEY POINTS **QUESTION:** Can PET/CT assess [^89^Zr]Zr-oxine–labeled human NK cell infiltration in HER2-positive breast tumors and quantify whether HER2-targeted trastuzumab therapy enhances this infiltration? **PERTINENT FINDINGS:** ^89^Zr-NK cells migrate to orthotopic HER2-expressing HCC1954 human tumors, with enhanced infiltration facilitated by HER2-targeted trastuzumab at early time points, aligning with clinical evidence. **IMPLICATIONS FOR PATIENT CARE:** The use of ^89^Zr and PET/CT can aid the development and understanding of antibody therapies, in the context of the immune environment and Fc-mediated therapeutic effects.
